# Convalescence via critical care collaboration

**DOI:** 10.1186/cc13203

**Published:** 2014-03-17

**Authors:** C Shute, A Saayman

**Affiliations:** 1 University Hospital Wales, Cardiff, UK

## Introduction

A collaborative approach to patient management has been shown to improve patient outcomes. In a resource-limited NHS, critical care beds are at a premium, therefore preventing unnecessary admissions by optimising ward-based management is essential. The objective of this service quality improvement project was to improve collaboration between the critical care directorate and neurosurgical high care unit at a tertiary university teaching hospital. We proposed that the use of a simple ward round tool on collaborative rounds would facilitate systematic patient review, prompt early recognition of those critically unwell and improve patient outcomes.

## Methods

An initial observation period of behaviours and practice on the neurosurgical unit was conducted with qualitative and quantitative data collection. Following analysis of these outcome measures a simple ward round tool was constructed, with the mnemonic ‘DON'T FORGET', and we devised a programme for collaborative ward rounds to take place three times per week over a 1-month period. During this time further data were collected to assess whether our interventions resulted in modified behaviours and to document the number of changes made to patient management as a consequence of the collaborative approach to care.

## Results

Our results showed that improvements were made in all assessed domains. Consultant-led ward rounds increased, attendance by members of the multidisciplinary team (MDT) dramatically improved and as a result MDT discussion was enhanced. In addition, documentation of structured plans improved and review of prescription charts significantly increased. As a consequence of collaborative rounds, a total of 343 changes were made to patient management under the domains of the ward round tool. This was an average of 21.4 changes per collaborative round, with most changes being made to medication charts or decisions regarding thromboprophylaxis.

## Conclusion

The use of a simple ward round tool combined with a collaborative approach to ward rounds improved MDT involvement and discussion, promoted structured patient review and resulted in positive changes to multiple areas of patient management. In conclusion, structured collaborative rounds result in significant changes to patient management that may prevent admission, or readmission, to critical care which has the potential to reduce healthcare costs and morbidity.

